# Private and social time preference for health outcomes: A general population survey in Iran

**DOI:** 10.1371/journal.pone.0211545

**Published:** 2019-02-01

**Authors:** Alireza Mahboub-Ahari, Abolghasem Pourreza, Ali Akbari Sari, Trevor A. Sheldon, Maryam Moeeni

**Affiliations:** 1 Department of Health Economics, and Health Management Research Center, Tabriz University of Medical Sciences, Tabriz, Iran; 2 Department of Health Management and Economics, and Knowledge Utilization Research Center, Tehran University of Medical Sciences, Tehran, Iran; 3 Department of Health Sciences, University of York, Heslington, York, United Kingdom; 4 Health Management and Economics Research Center, Isfahan University of Medical Sciences, Isfahan, Iran; Middlesex University, UNITED KINGDOM

## Abstract

Despite the recent increase in economic evaluations of health care programs in low and middle income countries, there is still a surprising gap in evidence on the appropriate discount rate and the discounting of health outcomes such as quality adjusted life years (QALYs). Our study aimed to calculate the implied time preference rate for health outcomes in Iran and its key determinants. Data were gathered from one family member from each of the 650 households randomly selected in Tehran. The respondents’ private and social preferences for health outcomes were calculated using the time trade-off (TTO) technique based on the discounted utility model. We investigated the main assumptions of the discounted utility model through equality of mean comparison, and the association between private time preference and key socio-economic determinants using multilevel regression analysis. The mean and median implied rates were 5.8% and 4.9% for private time preference and 25.6% and 20% for social time preference respectively. Our study confirmed that magnitude, framing and time effects have a significant impact on implied discount rates, which means that the conventional discounted utility model’s main assumptions are violated in the Iranian general population. Other models of discounting which apply lower rates for far health outcomes might provide a more sensible solution to discounting health interventions with long-term impacts.

## Introduction

The choice of discount rate as well as the practice of discounting health outcomes influences the decision-making process when informed by an economic evaluation [1, 2]. Despite the relative abundance of published studies about discount rate estimation and discounting practice in high income countries (HICs), [3] there is a significant paucity of literature in this regard in low and middle income countries (LMICs), particularly with respect to health outcomes. However, growing attention has been paid to social preferences and investment cases. For example studies have found that people in India are in general more likely than Americans to be classified as spiteful and less likely to be classified as altruistic, cooperative and socially efficient than US residents [4–6]. The choice of appropriate discount rates for LMICs is particularly challenging due to greater market imperfections, varying inter-generational weighting and values, political instability and cultural factors [7, 8].

A recent systematic review [9] found only two [7, 10] population-based time preference studies undertaken in LMICs investigating general public preferences about health outcomes. This gap in evidence on appropriate discount rates and discounting practice for health related outcomes is surprising given the recent increase in economic evaluations of health care programs or health technology assessments (HTA) in LMICs [11–13].

Poulos et al. [10] estimated the social discount rate for health outcomes among samples of general population in Ethiopia, Bulgaria, Ukraine, Mozambique, Uganda and Indonesia. They elicited respondents’ time preferences about two lifesaving programs adopted from Cropper’s experiment which examined people social time preferences [14]. This study found that the median social discount rate varied widely between the countries, ranging from 15% in Mozambique to 20.1% in Ukraine. The researchers highlighted that, individuals in all the six countries were much more present-oriented (more heavily discounting the future) compared to their counterparts in HICs. They also concluded that the constant exponential discount function could not truly reflect respondents’ time preferences for saving lives. Although the multivariate regression analysis provided strong support for hypotheses regarding the effect of alternative health programs, they failed to find the expected income effect.

Despite the benefits of social time preference in discounting social health investments, this type of preference cannot mirror individuals’ preferences about their personal health, which is most applicable in discounting health outcomes [2]. Therefore, focusing on private time preferences, which address individuals’ preferences about their own health, appears to be more appropriate in discounting health outcomes and studying health affecting behaviors [15–18].

Robberstad et al compared estimated private and social discount rates in the general population of Tanzania using the stated preference approach. The implied mean and median discount rates for both private and social perspectives were very similar, estimated as 0.07 and 0.058 respectively [7].

The potential for generalizing population-based time preference findings from one country to another is limited. Robberstad et al [7] emphasized that their study’s results could not be transferred to other contexts, even to other African countries. Researchers have recommended that further investigation is needed to obtain more knowledge about private and social time preferences for own and others’ health in other countries.

This study is the first to estimate appropriate discount rates of health outcome for use in health care decision making in Iran. As the second largest economy in the Middle East and North Africa (MENA) region, spending 7% of its GDP on healthcare, Iran is an attractive market for foreign medical device and pharmaceutical products. Since the establishment of the Office of HTA in the Iranian Ministry of Health in 2007, requests for economic evaluation projects in general and technology appraisals in particular have been growing rapidly [19]. However, there is little consensus about discounting practice and the size of discount rate. We aimed to answer three questions: 1) what are the implied private and social time preference rates for health outcomes in the Iranian general population 2) how do implied time preference rates vary across different scenarios and socio-economic characteristics of the population, and 3) which discounting practice is appropriate for Iran?

## Materials and methods

We conducted a cross sectional structured interview survey during 2014–2015 with a random sample of general population in the capital city of Tehran (the most populous city of Iran, with a population of 8.8 million), using a stratified multistage cluster sampling design. We classified 21 municipal districts of the city into five social classes reported as: high, upper-middle, middle, lower-middle and low. Each of these classes represented a sampling stratum covering several districts with similar socio-economic characteristics. In the first stage, one district was selected from each stratum (five in total) through a simple random selection. In the second stage, the identity code and postal address of all the dwellings in each selected district were acquired from the Statistical Center of Iran, and 130 households were systematically drawn from each district. Finally, one family member aged over 18 who was able to respond to the questions from each of the 650 households, was selected as study participant. In order to increase sample representativeness, age and gender of the respondents were adjusted according to their distribution in the target population.

Data on individuals’ preferences were collected using a two-part structured questionnaire administered by face to face interview with the participants (see the supplementary information). The first part gathered data on respondents’ time preferences about private (own) health issues while the second part of the gathered data related to their preferences about societal health issues (as outlined below). To elicit time preferences, we applied the stated preferences approach, widely applied for valuing health outcomes, especially in the absence of actual markets. Interviewers explained pre specified inter-temporal choice scenarios to the respondents and asked them to choose the one they preferred.

Five professional interviewers received two training sessions on interview techniques and also conducted 10 practice interviews with a convenience sample of respondents and a pilot was conducted to assess issues with the administration of the survey. Verbal informed consent was obtained from the respondents before the interview. A 10% telephone check and quality control of the completed questionnaires was carried out. The study was approved by the Ethics Committee of Tehran University of Medical Sciences (reference 9201102–22344).

### Private time preference rates

In the first part of questionnaire, two familiar as well as non-fatal health state scenarios including “influenza like disease” and “disabling injury of car accident” were used as hypothetical health states. Then, the participants were asked to imagine being affected by a disease resulting in a state like those mentioned above. Thus, similar to Cairns [2], we adopted an 11221 EQ5D health state scenario (the influenza like disease) presenting no problems in walking about, no problems with self-care, some problems with performing usual activities, moderate pain or discomfort and not anxious or depressed. In order to test the magnitude effect, we also asked the respondents two questions with a relatively severe health state scenario that was a “disabling injury of car accident”. That health state represented having slight problems in all dimensions (i.e. a 22222 EQ5D health state).

For each scenario, two choice items were presented, A: X days of ill health in a point of time in the near future (t) and B: Y(Y>X) days of exactly the same ill health at some time in the far future (t+β). Then, they were asked which of A and B they preferred, and how long would Y have to be for them to be indifferent between the two choice items. Each respondent’s indifference point was determined based on the time trade-off approach. Finally, the mean, median and range as well as the 10th and 90th percentiles of the implied discount rates were calculated for each question through the discounting factor formula:$\;PV\;=\;SV({\frac{1}{1+r})}^{t}$, where PV is the number of days living in given health status at present time, SV is the respondent’s stated value for a proposed health state in the future, t is the time horizon (delay) and r is the discount rate. Immediate time was not used as the starting point to avoid the immediacy effect. In our experiment, the starting points were set as either 2 or 3 years after the time of the survey. Setting the start point in the future may result in lower discount rates relative to immediate points in time. In order to facilitate the comparison between inter-temporal values, the choice items were presented in a separate show card [2].

Table 1 shows the questions asked for eliciting private preference rates for health outcomes. A relatively wide range of time delays (2–13 years) was included in the choices [2]. Each respondent was presented with eight questions. The first six questions included the same health state, three with the starting point of two years and the rest with the starting point of three years after the survey time. For each starting point, three different delays of a short-term (2–5 years), a medium-term (6–9 years) and a long-term (10–13 years) were presented to respondents. Four versions of the questionnaire (V1, V2, V3 and V4) were therefore developed to distribute 12 different delays across six questions (Table 1). Different time delays and starting points facilitated the investigation of the time effect and testing of the stationarity assumption. The assumption of decreasing timing implies that individuals give lower discount rates for far-future events. The stationarity assumption implies that individuals’ time preference rates (r) are constant over time [3, 20, 21] and is accepted when the implied rates are identical for scenarios with the same health state and time delays but different starting points. The respondents were asked two additional questions with the same starting point and time delay as for the first two questions in each version of the questionnaire, but with the more severe health state (22222 versus 11221). Since the first part of the questionnaire included only loss scenarios, we could not test the framing effect for private preferences.

**Table 1:** Design of the study questions for eliciting private time preference rates.

Question (health State)	Starting point	Delay			
		V1	V2	V3	V4
***Q1(11221)***	*2*	*2*	*3*	*4*	*5*
***Q2(11221)***	*2*	*7*	*9*	*6*	*8*
***Q3(11221)***	*2*	*10*	*11*	*12*	*13*
***Q4(11221)***	*3*	*3*	*5*	*2*	*2*
***Q5(11221)***	*3*	*6*	*7*	*8*	*9*
***Q6(11221)***	*3*	*10*	*11*	*12*	*13*
***Q7(22222)***	*2*	*2*	*3*	*4*	*5*
***Q8(22222)***	*2*	*7*	*9*	*6*	*8*

22222: some problems in walking about, some problems with self-care, some problems with performing usual activities, moderate pain or discomfort and moderate anxious or depressed.

### Social time preference rates

In the second part of the questionnaire, six questions were designed to elicit social time preference rates; three presented a gain scenario (saving lives through a health program such as a government sponsored local immunization program or a local water sanitation program) and the rest presented a loss scenario (postponement of death due to an airplane crash). Thus, gain/loss scenarios only occurred in the social time preference questions. Such questions yield the chance of testing framing effect which we could not perform for private preferences. As shown in Table 2, a wide range of time delays and a magnitude of gain/loss event were presented across the four versions of the study questionnaire [15, 22, 23].

**Table 2:** Design of the study questions for eliciting social time preference rates.

Question (scenario)	Starting point	Delay			
		V1	V2	V3	V4
***Q1(1000 lives saved)***	2	1	2	3	4
***Q2(10 lives lost)***	2	1	2	3	4
***Q3(10000 lives saved)***	2	1	2	3	4
***Q4(100 lives lost)***	2	1	2	3	4
***Q5(1000 lives saved)***	2	6	7	8	9
***Q6(10 lives lost)***	2	6	7	8	9

In order to test the main assumptions of the discounted utility model in social preferences, the mean comparison of the elicited time preference rates was carried out among different questions. Depending on the normality test result, an appropriate equality of mean comparison test was used to compare the elicited social discount rates. For example, the magnitude effect was tested through comparing the mean implied rates for questions 1 with 3, and question 2 with 4. The time effect was tested through comparing the implied rates for question 1 (gain with 2–5 years delay) with 5 (gain with 10–13 years delay) and 2 (loss with 2–5 years delay) with 6 (loss with 10–13 years delay).

### Econometric model for private discount rates

A multilevel random intercept model was estimated to study the hierarchical effect of the variables of interest related to the scenarios and respondents on the elicited private discount rates for health outcomes. The model can be understood as a two-level model with scenarios as the first level and respondents as the second level. There were eight observations per respondent at the first level. The private discount rate was the dependent variable, which was regressed on the independent variables of interest including: time delay (which ranged from 2 to 13 years) and three indicator variables. The first one captured whether the starting point was 3 years, the second measured whether the health state was 22222 of the EQ5D health states and the third indicator variable captured the four versions of the questionnaire. At the second level, the respondents’ characteristics included: age (<25, >25<65, >65), gender, marital status (single, married, and widow), education level (whether they were educated beyond secondary school), employment status, smoking status, social class, home ownership, and experience of chronic disease. Finally, the interaction terms between the variables were included in the model.

The analysis was conducted in Stata 11 (Stata Corp. 2009. Stata Statistical Software: Release 11. College Station, TX: Stata Corp LP.) And MLwiN 2.33 (Bristol University, Bristol).

## Results

There was an overall 93% response rate, which varied by question; a total of 606 completed questionnaires were analyzed. The age and gender distribution in the study samples was similar to that of the whole population, according to the last census of the Iranian households in 2011. Characteristics of the sample are summarized in Table 3.

**Table 3:** Characteristics of the study sample (N = 606).

Variable	N (%)
**Age (year)**	
***18–24***	112 (18.5)
***25–34***	167 (27.6)
***35–44***	118 (19.5)
***45–54***	93 (15.3)
***55–64***	70 (11.6)
***+65***	46 (7.6)
**Gender**	
***Male***	304 (50.2)
***Female***	302 (49.8)
**Marital Status**	
***Single***	161 (27)
***Married***	411 (68)
***Widow***	34 (5)
**Working Status**	
*Full time*	91(15)
***Part time***	142 (23)
***Unemployed***	12 (2)
***Retired***	70 (12)
***Housewife***	178 (29)
***Other***	111 (19)
**Education attainment**	
***Illiterate***	18 (3)
***Up to high School***	303 (50)
***Undergraduate***	210 (35)
***Postgraduate***	75 (12)
**Social class**	
*High (fifth quintile)*	121 (20)
*Upper-middle (fourth quintile)*	121 (20)
*Middle (third quintile)*	123 (20.5)
*Lower-middle (second quintile)*	121 (19.8)
*Low (first quintile)*	120 (19.7)

### Private and social time preference rates

The implied time preference rates derived according to each question of the private and social scenarios are summarized in Table 4. The time preference rates ranged from 55% to 117% for private health outcomes and 0% to 116% for social health outcomes. There were wide variations in the time preference rates in terms of the scenario type, start point (SP), proposed delay (Del), magnitude of events, and respondents’ characteristics. Since the negative time preference rates have been justified in previous studies [1–3, 24, 25], we included negative and zero preferences in calculating the mean rate.

**Table 4:** The implied private and social time preference rates.

Question Detail	N	Mean	Median	Min	Max	Percentile	
**Private Time Preferences (rate)**						10	90
*Q1 (Scenario*: *11221*, *Starting Point*: *2*, *Delay*: *2–5 year)*	606	9.4	7.7	-52.7	108	0	26
*Q2 (Scenario*: *11221*, *Starting Point*: *2*, *Delay*: *6–9 year)*	605	4.5	4.5	-23.5	34.8	-0.7	10.7
*Q3 (Scenario*: *11221*, *Starting Point*: *2*, *Delay*: *10–13 year)*	604	3.1	3.4	-17.5	20.1	-0.9	7.7
*Q4 (Scenario*: *11221*, *Starting Point*: *3*, *Delay*: *2–5 year)*	603	11	8.6	-55.3	61.2	0	31.1
*Q5 (Scenario*: *11221*, *Starting Point*: *3*, *Delay*: *6–9 year)*	603	4.2	4	-23.3	25.1	-0.9	10.2
*Q6 (Scenario*: *11221*, *Starting Point*: *3*, *Delay*: *10–13 year)*	603	2.8	3.5	-19	25	-1.7	7
*Q7 (Scenario*: *22222*, *Starting Point*: *2*, *Delay*: *2–5 year)*	603	8	4.9	-43.8	58.1	-2.1	24.5
*Q8 (Scenario*: *22222*, *Starting Point*: *2*, *Delay*: *6–9 year)*	603	3.5	3.3	-31.9	117.5	-0.9	16.2
**Social Time Preferences (rate)**							
*Q1 (Scenario*: *gain*, *Delay*: *2–5 years*, *magnitude*: *1000)*	600	46.6	37.5	0	210	5.1	100
*Q2 (Scenario*: *loss*, *Delay*: *2–5 years*, *magnitude*: *10)*	598	18.3	12.5	0	115.4	0	60
*Q3 (Scenario*: *gain*, *Delay*: *2–5 years*, *magnitude*: *10000)*	598	35.3	22.5	0	200	4.9	90
*Q4 (Scenario*: *loss*, *Delay*: *2–5 years*, *magnitude*: *100)*	606	16.1	11	0	115.6	0.3	51
*Q5 (Scenario*: *gain*, *Delay*: *10–13 years*, *magnitude*: *1000)*	596	26.7	28.5	0	72.9	7.9	41.4
*Q6 (Scenario*: *loss*, *Delay*: *10–13 years*, *magnitude*: *10)*	595	10.7	9.6	0	59.2	3.8	17.5

### Magnitude and time effect of social time preference

The mean comparison test supported the existence of a magnitude effect, meaning that respondents discounted social health outcomes of greater magnitude with lower discount rates. Our analysis also demonstrated decreasing timing aversion, meaning that the respondents had lower implied discount rates for distant health outcomes (Table 5).

**Table 5:** The magnitude and time effect in the social time preference rates.

Assumption	Mean Comparison test		Test Result
*Magnitude Effect*	*The mean discount rate for question 1*	*The mean discount rate for question 3*	t = 44.2 P <0.001
	*The mean discount rate for question 2*	*The mean discount rate for question 4*	t = 5.9 P <0.001
*Time effect*	*The mean discount rate for question 1*	*The mean discount rate for question 5*	t = 34.7 P <0.001
	*The mean discount rate for question 2*	*The mean discount rate for question 6*	t = 25.4 P <0.001

### Factors affecting private time preference rate

Potential determinants of the private time preference rates were investigated through multilevel analysis. Variables entered in the model at first level included the scenario type, delay (ranging from 2 to 13 years), version of questionnaire (the four versions), and starting point (2 or 3 years). Variables entered in the model at second level were personal characteristics including age, gender, marital status, education level, residential status, etc. The regression analysis of the private time preference rates is presented in Table 6; all the estimated results are based on the assumption of linear utility.

**Table 6:** The factors affecting the private time preference rate.

Variable	Coefficient	P-Value
**Delay**	-0.96	**001**
*Scenario of “disabling injury of car accident”*	-1.87	**001**
*Start point as 3 years from survey time*	0.16	**0.49**
**Version of questionnaire**		
*V1*	1.35	**0.157**
*V2*	0.33	**0.729**
*V3*	0.91	**0.389**
*V4*	-	**-**
*Over 65 years old*	-0.29	**0.899**
*Under 25 years old*	3.25	**0.051**
*Female*	-0.76	**0.433**
**Marital status:**		
*Single*		
*Married*	5.67	**0.005**
*Widow*	5.87	**0.008**
*Housewife*	-2.12	**0.193**
*Living in female headed household*	-1.97	**0.249**
*Diploma*	-0.09	**0.915**
*University degree (undergraduate or postgraduate)*	-0.70	**0.417**
*Smoker*	-1.55	**0.0743**
*Living in a home owner household*	-1.43	**0.022**
*Experiencing acute diseases*	-0.45	**0.728**
*Experiencing chronic diseases*	-14.37	**0.0286**
In first quintile of social class* Experiencing acute diseases	-2.71	**0.0883**
*In first quintile of social class*	-0.84	**0.581**
*In third quintile of social class*	-1.00	**0.532**
*In fourth quintile of social class*	-1.37	**0.364**
In fifth quintile of social class * Experiencing chronic diseases	20.80	**0.003**
In fourth quintile of social class * Experiencing chronic diseases	13.700	**0.07**
Married * Under 25 years old	0.280	**0.908**
Married * Over 65 years old	-3.56	**0.057**
In third quintile of social class * Employed	-3.40	**0.06**
In second quintile of social class * Employed	-2.60	**0.122**
In fifth quintile of social class * Employed	-2.90	**0.101**
***συ*** *(Standard deviation between respondents)*	38(2.6)	
***σɛ*** *(Standard deviation between questionnaire versions*)	46 (1.0)	

There was a significant negative association between the time delay and time preference rates and thus the respondents discounted distant events with lower rates (decreasing timing aversion) [26]. Similarly, the negative association between the scenario type and implied rates confirmed the existence of a magnitude effect, where people applied lower discount rates for more severe health outcomes. There was no significant difference between the imputed time preference rates using different versions of the questionnaire, implying that applying a wide range of time delays in the four versions of the questionnaire did not bias the results. The estimated rate for two starting points was the same and also the estimated coefficient for the starting point of 3 years was not significant, indicating that the assumption of stationarity is accepted. In other words, the respondents’ preferences did not vary when the starting point was delayed by a year.

With regard to the demographic characteristics of respondents, both the married and widowed respondents tended to discount future health outcomes more heavily than those who were single. Homeowners were more far-sighted than tenants; home ownership is a proxy for economic status, which directly increases access to healthcare in Iran. Respondents’ experience of a long-term illness appeared to have a significant influence on implied rates, resulting in lower rates for all of the scenarios. We found no significant relationship between time preference rates and respondents’ age, gender, employment status, smoking, education level, and social class.

## Discussion

This population-based study is the first in Iran to estimate time preference rates for private and social health outcomes. All private rates pertain to loss scenarios but social rates pertain to both loss and gain scenarios. We used a stated preference approach, the mean time preference rates for all the questions were calculated by using common discount function. In order to test the main assumptions of the discounted utility model, we used the mean comparison test for different magnitudes and time delays. The key influential factors affecting private time preferences were examined using a multilevel regression analysis, in which two ill health states (losses), wide range of delay (2–13 years), and two start points were adopted to facilitate the experiment.

The literature shows great heterogeneity among empirical studies in terms of context, study scenario, time span and magnitude of proposed outcomes, which has caused high degree of variation in estimated time preference rates [1, 2, 7, 10, 15, 26–28]. In this study, the mean implied time preference rate for private health outcomes was estimated as 5.8% while the rate estimated by previous studies which were mostly conducted in high income countries ranged from 1% to 46%. Similarly, the mean implied rate for social health outcome was 25.6% which lays among the rates estimated in previous empirical studies [9]. It means that the estimated rates for both private and social health outcomes are reasonably in line with previous empirical studies.

We showed that from a total 4873 implied time preference rates, 1133 (23%) were zero or negative. This implies that a significant proportion of society gives no value to postponing health problems and proves the existence of negative time preferences in the Iranian population. This supports Cairn’s [2], Lowenstein’s [3], Ganiat’s [29] and West’s [15] conclusions about this type of preference that is revealed when individuals perceive future events to be more uncertain and dreadful. More careful analysis is needed to better understand why some people prefer to sacrifice their good health now in return for poor health in the future or to assess whether this is a reflection of poor understanding of the questions or the exercise as a whole.

The significant difference between mean social discount rates with regard to different magnitudes and time delays show a magnitude effect and decreasing timing aversion. These both suggest violation of the discounted utility model’s main assumptions examined in previous work [2, 3, 21].

The regression analysis showed that Iranians’ discounting behaviour varied significantly across different time delays (time effect) and severity of health state (magnitude effect). This strongly violates the assumption of constant timing aversion and instead supports the decreasing timing aversion, meaning that individuals’ timing behaviour can be explained better by hyperbolic discounting models than by the conventional discounted utility model. This finding is in agreement with the majority of previous research [7, 14, 22, 28–32], which also show ‘delay speed-up asymmetry’ for explaining the effect of time delay on time preference rates [22].

The regression analysis confirmed the correlation between private time preference rate and some factors previously shown in the literature [1, 2, 7] but contradicted others [14, 33, 34]. Given the high degree of heterogeneity in the respondents’ time preference rates and their understanding of the questions, this is perhaps not surprising. Therefore, more experiments with larger sample sizes and a wider variety of delays, scenarios and questions might yield more information about the real range of individual time preferences [1].

Despite the abundance of theoretical and empirical work on discounting practice, the appropriateness of using a stated preference approach as a basis for adjusting future costs and benefits remains debatable. According to the literature from both HICs and LMICs, the estimated rates from individuals’ time preferences might not provide solid evidence, on which to firmly base the practice of discounting future health outcomes. Therefore, in HICs, despite the existence of empirical studies reporting high discount rates, the practice is generally to use a lower rate of around 3–5% for both health and monetary outcomes. Bobinac et al. [35] and West et al. [15] also concluded that more caution is necessary when applying estimated discount rates. Severens et al. stated that individuals’ preferences are hard to apply generally for discounting future health benefits because they are very situation specific [36].

In order to derive an evidence-based consensus on appropriate discounting practice, another suggested approach is to estimate social time preference rates through the revealed preference approach which mirrors society’s decisions in the real world rather than being based on individuals’ preferences about hypothetical conditions. According to this perspective, discounting practice should be based on the opportunity costs of financing health care [37, 38]. This is because the health care costs of a project could have been invested elsewhere in the economy or used to reduce public borrowing at a real rate of return, which would provide more health care resources in the future and generate greater health benefits. If health care costs are discounted to reflect the opportunity cost of financing health care, their health effects must be discounted at the same rate.

We used a comprehensive questionnaire with wide range of delays and multiple scenarios which yielded the chance of testing several hypotheses. However, the study faces some limitations. One of the limitations of our study is that it took place during a period of significant uncertainty around the presidential election result, economic instability and rising healthcare costs. These might have biased the results towards higher implied rates. It is possible that there was a question order effect (questions to elicit private rates before those eliciting social rates). In future, this could be randomly ordered and any order effect could be formally tested. The other limitation should be mentioned is that private time preference questions and the social time preference questions involve different scenarios which limit their comparability. The questionnaire we developed to collect data for the study subjects included about more than 26 questions. We believed that asking further questions may be frustrating for both interviewer and respondents.

Due to several Iranian contextual factors, mainly presidential election, economic instability and rapid rising costs, the estimated time preference rates in our survey should not be directly used as a discount rate for health outcomes. However, the results of hypothesis testing which favored decreasing timing aversion can enhance researchers’ understanding about individuals’ time preferences and inform the practice of discounting in economic evaluations and health technology assessments. Given the lack of scientific evidence, we suggest that application of the recommended discount rate by organizations such as World Bank, World Health Organization (WHO), and National Institute for Care Excellence (NICE) [39, 40] can be adopted by researchers in Iran and similar settings as a temporary measure.

## Conclusion

Our study confirmed that the main assumptions of the conventional discounted utility model were violated. Other models of discounting, which apply lower rates for far health outcomes, might provide a more sensible solution to discounting health interventions with long-term impacts. The Health Technology Assessment, Standardization and Tariffs Office at the Iranian Ministry of Health needs to officially announce its policies about appropriate discount rate and discounting practice, as many studies conducted in the Iranian context need discounting as one of the main parts of their analyses.

## Supporting information

S1 Dataset (SAV)

S1 Questionnaire (DOCX)

## References

[1] van der Pol M, Cairns J, van der Pol M, Cairns J. Comparison of two methods of eliciting time preference for future health states. Social Science & Medicine. 2008;67(5):883–9. 10.1016/j.socscimed.2008.05.011 . 18571298

[2] Cairns JA, van der Pol MM. The estimation of marginal time preference in a UK-wide sample (TEMPUS) project. Health Technol Assess. 2000;4(1):i–iv, 1–83. . 10682274

[3] Loewenstein G, Prelec D. Anomalies in Intertemporal Choice: Evidence and an Interpretation. The Quarterly Journal of Economics. 1992;107(2):573–97.

[4] Capraro V, Corgnet B, Espín AM, Hernán-González R. Deliberation favours social efficiency by making people disregard their relative shares: evidence from USA and India. Royal Society open science. 2017;4(2):160605 10.1098/rsos.160605 28386421 PMC5367314

[5] Capraro V, Cococcioni G. Social setting, intuition and experience in laboratory experiments interact to shape cooperative decision-making. Proc R Soc B. 2015;282(1811):20150237 10.1098/rspb.2015.0237 26156762 PMC4528537

[6] Capraro V, Jordan JJ, Rand DG. Heuristics guide the implementation of social preferences in one-shot Prisoner’s Dilemma experiments. Scientific reports. 2014;4:6790 10.1038/srep06790 25348470 PMC4210943

[7] Robberstad B, Robberstad B. Estimation of private and social time preferences for health in northern Tanzania. Social Science & Medicine. 2005;61(7):1597–607. 10.1016/j.socscimed.2005.03.013 . 15885866

[8] Robberstad B, Cairns J, Robberstad B, Cairns J. Time preferences for health in northern Tanzania: an empirical analysis of alternative discounting models. Pharmacoeconomics. 2007;25(1):73–88. 10.2165/00019053-200725010-00007 . 17192119

[9] Mahboub-Ahari A, Pourreza A, Akbari Sari A, Rahimi Foroushani A, Heydari H. Stated Time Preferences for Health: A Systematic Review and Meta Analysis of Private and Social Discount Rates 2014. 25209903

[10] Poulos C, Whittington D. Time Preferences for Life-Saving Programs: Evidence from Six Less Developed Countries. Environmental Science & Technology. 2000;34(8):1445–55. 10.1021/es990730a

[11] Santatiwongchai B, Chantarastapornchit V, Wilkinson T, Thiboonboon K, Rattanavipapong W, Walker DG, et al Methodological Variation in Economic Evaluations Conducted in Low- and Middle-Income Countries: Information for Reference Case Development. PLOS ONE. 2015;10(5):e0123853 10.1371/journal.pone.0123853 25950443 PMC4423853

[12] Asante A, Price J, Hayen A, Jan S, Wiseman V. Equity in Health Care Financing in Low- and Middle-Income Countries: A Systematic Review of Evidence from Studies Using Benefit and Financing Incidence Analyses. PLOS ONE. 2016;11(4):e0152866 10.1371/journal.pone.0152866 27064991 PMC4827871

[13] Horton S, Gelband H, Jamison D, Levin C, Nugent R, Watkins D. Ranking 93 health interventions for low- and middle-income countries by cost-effectiveness. PLOS ONE. 2017;12(8):e0182951 10.1371/journal.pone.0182951 28797115 PMC5552255

[14] Cropper M, Aydede S, Portney P. Preferences for life saving programs: how the public discounts time and age. Journal of Risk and Uncertainty. 1994;8(3):243–65.

[15] West RR, McNabb R, Thompson AG, Sheldon TA, Grimley Evans J, West RR, et al Estimating implied rates of discount in healthcare decision-making. Health Technology Assessment (Winchester, England). 2003;7(38):1–60. . 14622489 10.3310/hta7380

[16] Parsonage M, Neuburger H, Parsonage M, Neuburger H. Discounting and health benefits. Health Economics. 1992;1(1):71–6. . 1342633 10.1002/hec.4730010110

[17] Goto R, Takahashi Y, Nishimura S, Ida T, Goto R, Takahashi Y, et al A cohort study to examine whether time and risk preference is related to smoking cessation success. Addiction. 2009;104(6):1018–24. 10.1111/j.1360-0443.2009.02585.x . 19466926

[18] Cadier B, Durand-Zaleski I, Thomas D, Chevreul K. Cost Effectiveness of Free Access to Smoking Cessation Treatment in France Considering the Economic Burden of Smoking-Related Diseases. PLOS ONE. 2016;11(2):e0148750 10.1371/journal.pone.0148750 26909802 PMC4766094

[19] Doaee SH, Olyaeemanesh A, Emami SH, Mobinizadeh M, Abooee P, Nejati M, et al Development and Implementation of Health Technology Assessment: A Policy Study. Iranian Journal of Public Health. 2013;42(Supple1):50–4. 23865016 PMC3712594

[20] Halevy Y. Time Consistency: Stationarity and Time Invariance. Econometrica. 2015;83(1):335–52. 10.3982/ECTA10872

[21] Bleichrodt H, Johannesson M. Time Preference for Health: A Test of Stationarity versus Decreasing Timing Aversion. J Math Psychol. 2001;45(2):265–82. 10.1006/jmps.2000.1312 . 11302713

[22] Lazaro A, Barberan R, Rubio E, Lazaro A, Barberan R, Rubio E. Private and social time preferences for health and money: an empirical estimation. Health Economics. 2001;10(4):351–6. 10.1002/hec.599 . 11400257

[23] Cairns JA, Cairns JA. Valuing future benefits. Health Economics. 1994;3(4):221–9. . 7994322 10.1002/hec.4730030404

[24] Van Der Pol MM, Cairns JA, Van Der Pol MM, Cairns JA. Negative and zero time preference for health. Health Economics. 2000;9(2):171–5. . 10721018 10.1002/(sici)1099-1050(200003)9:2<171::aid-hec492>3.0.co;2-z

[25] Andersen S, Harrison GW, Lau MI, Rutström EE. Eliciting Risk and Time Preferences Econometrica. 2008;76(3):583–618.

[26] Cairns J, van der Pol M. Constant and decreasing timing aversion for saving lives. Soc Sci Med. 1997;45(11):1653–9. Epub 1998/01/15. . 9428085 10.1016/s0277-9536(97)00100-7

[27] Cropper MLA, Sema K & Portney Paul R. Rates of Time Preference for Saving Lives. The American Economic Review. 1992;82(2):469–72.

[28] Olsen JA. Time preferences for health gains: an empirical investigation. Health Econ. 1993;2(3):257–65. Epub 1993/10/01. . 8275171 10.1002/hec.4730020309

[29] Ganiats TG, Carson RT, Hamm RM, Cantor SB, Sumner W, Spann SJ, et al Population-based time preferences for future health outcomes. Medical Decision Making. 2000;20(3):263–70. 10.1177/0272989X0002000302 . 10929848

[30] Olsen JA, Olsen JA. On what basis should health be discounted? Journal of Health Economics. 1993;12(1):39–53. . 10126489 10.1016/0167-6296(93)90039-h

[31] Redelmeier DA, Heller DN, Redelmeier DA, Heller DN. Time preference in medical decision making and cost-effectiveness analysis. Medical Decision Making. 1993;13(3):212–7. 10.1177/0272989X9301300306 . 8412549

[32] Thaler R. Some empirical evidence on dynamic inconsistency. Economics Letters. 1981;8(3):201–7. 10.1016/0165-1765(81)90067-7.

[33] Golsteyn BHH, Grönqvist H, Lindahl L. Adolescent Time Preferences Predict Lifetime Outcomes. The Economic Journal. 2014;124(580):F739–F61. 10.1111/ecoj.12095

[34] Sozou PD, Seymour RM, Sozou PD, Seymour RM. Augmented discounting: interaction between ageing and time-preference behaviour. Proceedings of the Royal Society of London—Series B: Biological Sciences. 2003;270(1519):1047–53. 10.1098/rspb.2003.2344 . 12803894 PMC1691344

[35] Bobinac A, Brouwer W, van Exel J, Bobinac A, Brouwer W, van Exel J. Discounting future health gains: an empirical enquiry into the influence of growing life expectancy. Health Economics. 20(1):111–9. 10.1002/hec.1578 . 20029933

[36] Severens JL, Milne RJ. Discounting Health Outcomes in Economic Evaluation: The Ongoing Debate. Value in Health. 2004;7(4):397–401. 10.1111/j.1524-4733.2004.74002.x 15449631

[37] Paulden M, Claxton K, Paulden M, Claxton K. Budget allocation and the revealed social rate of time preference for health. Health Economics. 21(5):612–8. 10.1002/hec.1730 . 21438069

[38] Claxton K, Paulden M, Gravelle H, Brouwer W, Culyer AJ, Claxton K, et al Discounting and decision making in the economic evaluation of health-care technologies. Health Economics. 20(1):2–15. 10.1002/hec.1612 . 21154521

[39] Baltussen RM, Adam T, Tan-Torres Edejer T, Hutubessy RC, Acharya A, Evans DB, et al Making choices in health: WHO guide to cost-effectiveness analysis. WHO: WHO; 2003.

[40] Earnshaw J, Lewis G. NICE Guide to the Methods of Technology Appraisal. PharmacoEconomics. 2008;26(9):725–7. 10.2165/00019053-200826090-00002 18767892

