# Supplementary material for: Private and social time preference for health outcomes: A general population survey in Iran
Source: PLoS One. 2019 Feb 1;14(2):e0211545. doi: 10.1371/journal.pone.0211545 (PMC6358076; doi:10.1371/journal.pone.0211545)
Supplement: S1 Questionnaire — (DOCX) [file pone.0211545.s002.docx]

**Interview Schedule for Estimating Time Preference Rates for Health outcomes**

***Interviewer must explain the aim of the interview and take consent for participation, the presents the whole process of interview as below:***

This interview schedule contains 8 questions which involve making ***choices*** between two events (ill health state) happening in different points in time (***near*** and ***far*** future). Please kindly consider the two choices and decide whether the event happening in ***earlier time*** (a) is better or worse than the event happening at ***later time*** (b) in the future? Then we ask you to determine a value that makes you ***indifferent*** between near and far future.

***Interviewer firstly complete personal and socio-economic information section, then presents the health state 11221 catalog and makes sure that respondents could understand the health state***

**Sample Question for Private Time Preferences**

**Question 1**:

(Interviewer): Imagine a certain disease that may trouble you. Imagine that the disease is moderately uncomfortable that gives ***headache and pain in muscles and joints*** and you have ***some problems in usual activities***. You have ***no problems in walking about*** and ***doing daily activities***, you have ***no problems with self-care***, and ***you are not anxious or depressed***. One may say that this is a condition that in several ways ***resembles Flu***. Imagine that the disease ***starts after 2 years and lasts 20 days*** (a), if there was a single treatment that makes it possible to postpone the disease which ***starts after 4 years from now and lasts 30 days*** (b)***.*** Which one do you prefer***?***

***Show Flash Cards***: 20 days in 2 years? Or 30 days in 4 year?

20 days in 2 years

***Interviewer decreases the number of days until the respondent’s become indifferent between two options.***

30 days in 4 year?

***Interviewer increases the number of days until the respondent’s become indifferent between two options.***

How many days of disease makes (a) and (b) equivalent?

………….. Days

**Sample Question for Private Time Preferences**

(Interviewer): Suppose that The Ministry of Health is planning to invest on ***a healthcare program*** (for example vaccination, blood pressure control, etc.). The Ministry of Health has ***two*** alternative programs that cost the same, but that there was only available budget for one of them. We would like to ask you **which one of these programs you would choose**:

***Show Flash Cards:***

Program A would save 1000 lives after 2 years

or

Program B would save 1500 lives after 3 years

The answer:

Program A would save 1000 lives after 2 years

***Interviewer increases the number of lives until the respondent’s become indifferent between program A and B.***

Program B would save 1500 lives after 3 years

***Interviewer decreases the number of lives until the respondent’s become indifferent between program A and B.***

How many lives would make you indifferent between program A and B?

…………… Lives
